# Impact of HPMCAS on the Dissolution Performance of Polyvinyl Alcohol Celecoxib Amorphous Solid Dispersions

**DOI:** 10.3390/pharmaceutics12060541

**Published:** 2020-06-11

**Authors:** Marius Monschke, Karl G. Wagner

**Affiliations:** Department of Pharmaceutical Technology and Biopharmaceutics, University of Bonn, 53121 Bonn, Germany; marius.monschke@uni-bonn.de

**Keywords:** amorphous solid dispersion, hot-melt extrusion, dissolution, polyvinyl alcohol, HPMCAS, celecoxib

## Abstract

Amorphous solid dispersions (ASDs) have been proven to increase the bioavailability of poorly soluble drugs. It is desirable that the ASD provide a rapid dissolution rate and a sufficient stabilization of the generated supersaturation. In many cases, one polymer alone is not able to provide both features, which raises a need for reasonable polymer combinations. In this study we aimed to generate a rapidly dissolving ASD using the hydrophilic polymer polyvinyl alcohol (PVA) combined with a suitable precipitation inhibitor. Initially, PVA and hydroxypropylmethylcellulose acetate succinate (HPMCAS) were screened for their precipitation inhibitory potential for celecoxib in solution. The generated supersaturation in presence of PVA or HPMCAS was further characterized using dynamic light scattering. Binary ASDs of either PVA or HPMCAS (at 10% and 20% drug load) were prepared by hot-melt extrusion and solid-state analytics were conducted using differential scanning calorimetry (DSC), X-ray powder diffraction (XRPD) and fourier-transformed infrared spectroscopy (FT-IR). The non-sink dissolution studies of the binary ASDs revealed a high dissolution rate for the PVA ASDs with subsequent precipitation and for the HPMCAS ASDs a suppressed dissolution. In order to utilize the unexploited potential of the binary ASDs, the PVA ASDs were combined with HPMCAS either predissolved or added as powder and also formulated as ternary ASD. We successfully generated a solid formulation consisting of the powdered PVA ASD and HPMCAS powder, which was superior in monophasic non-sink dissolution and biorelevant biphasic dissolution studies compared to the binary and ternary ASDs.

## 1. Introduction

Amorphous solid dispersions (ASDs) represent a promising formulation approach in order to increase the bioavailability of poorly soluble drugs of the BCS class II [1,2,3,4]. Besides ensuring physical stability of the molecularly dispersed drug within the polymeric matrix [5], ASDs can exhibit two decisive features which are relevant for their performance. First, the generation of supersaturation in a decent amount of time upon dissolution i.e., dissolution rate [6] and second the ability to maintain supersaturation for a sufficient duration i.e., precipitation inhibition [7]. In many cases, these features cannot be accomplished by one single polymer. It is generally assumed that a high similarity regarding hydrophobicity between polymer and drug is beneficial in order to obtain a sufficient stabilization of the supersaturated drug in solution. Considering that poorly soluble drugs are basically of hydrophobic nature, the selection of a sufficient polymer would fall on one exhibiting hydrophobic properties, too [8]. It was shown that rather hydrophobic polymers in most cases exhibit a slower dissolution rate as compared to more hydrophilic polymers when formulated as ASD [9,10]. The phenomenon of amorphous-amorphous phase separation (AAPS) is another key factor that can alter the dissolution rate of an ASD. During AAPS the molecularly dispersed, single phased ASD separates into a drug-rich and a polymer-rich domain within the solid-state. This phenomenon is facilitated in presence of water (from adsorbed moisture or upon contact with the dissolution medium) as a result of enhanced molecular mobility due to the plasticization effect of water [11]. The occurrence of AAPS was shown to adversely influence the dissolution performance of ASDs [12,13]. Therefore it was shown that hydrogen bonds between drug and polymer are decisive in order to avoid AAPS upon contact with moisture [11,14,15]. On the other hand the presence of hydrogen bonds alone was often shown to be not sufficient to generate a satisfactory maintenance of the supersaturated state in solution upon dissolution of the ASD [16]. A few studies investigated ternary ASDs with a combination of hydrophilic and hydrophobic polymers and showed a benefit compared to the respective binary ASD formulation, by achieving optimized dissolution rate and supersaturation maintenance [17,18]. 

Celecoxib (CXB) is a compound with a very poor solubility (1.5 µg/mL) in aqueous medium at pH 6.8 and high hydrophobicity with a logP of 4 belonging to BCS class II [18,19]. Several studies have identified hydroxypropylmethylcellulose acetate succinate (HPMCAS), which is a rather hydrophobic polymer, as a well-suited precipitation inhibitor for supersaturated solutions of CXB [18,20,21]. In general, HPMCAS is a polymer which was, in many cases, successfully applied for the formulation of amorphous solid dispersions [22,23,24,25]. However, there is no report about a binary ASD consisting of CXB and HPMCAS, which reached as high a concentration as theoretically expectable based on the precipitation inhibitory potential. On the contrary, it was shown that ASDs consisting of CXB combined with a rather hydrophilic polymer like povidone or copovidone exhibited a fast dissolution rate but limited potential to maintain the generated supersaturation without addition of further precipitation inhibitors [26,27]. This proves the fact that the choice of suitable combinations of drug and polymer is complex and has to be carried out with care. 

Recently, a special grade of polyvinyl alcohol (PVA) for hot-melt extrusion purposes marketed as Parteck MXP^®^ was introduced by Merck [28,29]. This grade of PVA is of pronounced hydrophilic nature and is able to form hydrogen bonds, where the hydroxyl group can act as hydrogen donor and the carbonyl group (related to the unhydrolized acetate moieties) can act as hydrogen acceptor. These hydrogen bond sites could potentially interact with the hydrogen bond acceptor groups (S=O, CF or 2-N of the pyrazole ring) and the donor group (NH_2_), respectively. Therefore, it can be expected that an ASD comprising PVA and CXB would exhibit a high dissolution rate with a potentially low risk of AAPS upon contact with the dissolution medium. The aim of this study was to initially assess the potential of PVA and HPMCAS to stabilize the supersaturated state of CXB in solution. The generated supersaturation was characterized using dynamic light scattering (DLS), to gain a mechanistic insight. Subsequently, it was examined whether the results obtained from the supersaturation assay are applicable to the performance of binary ASDs. Finally, we aimed to combine a rapidly dissolving ASD with a strong precipitation inhibitor. In order to find a suitable concentration of the precipitation inhibitor, various concentrations of predissolved precipitation inhibitor were tested. Based on the required concentration a solid formulation was created, either by physical addition of the precipitation inhibitor or by preparation of a ternary ASD. The physical properties of the ASDs were investigated using X-ray powder diffraction (XRPD), differential scanning calorimetry (DSC) and fourier-transformed infrared spectroscopy (FT-IR). In order to investigate the dissolution behavior of the ASDs, the degree of swelling and erosion was monitored and compared to drug-free extrudates. The performance of the ASDs was tested by aid of biorelevant non-sink dissolution and biphasic dissolution studies. 

## 2. Materials and Methods 

### 2.1. Materials

**Figure 1:**
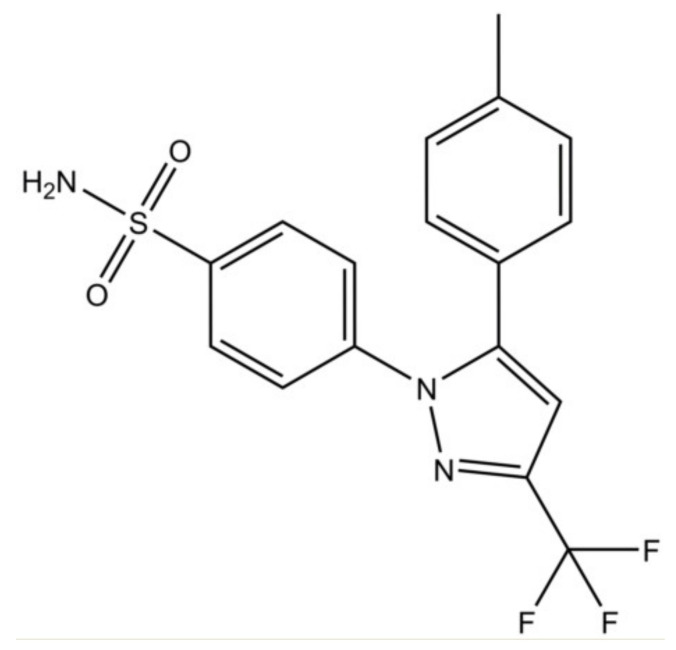
Chemical structure of celecoxib.

Polyvinyl alcohol (PVA, Parteck MXP^®^) was kindly donated by Merck (Darmstadt, Germany). HPMCAS LG (hydroxypropylmethylcellulose acetate succinate) was provided by Shin-Etsu Chemical (Tokyo, Japan). Copovidone, Kolliphor P188 and Kolliphor P407 were donated by BASF (Ludwigshafen, Germany). HPC-SSL was obtained from Nisso Chemical Europe (Düsseldorf, Germany) and Eudragit L100-55 was obtained from Evonik (Darmstadt, Germany). Celecoxib (CXB) was obtained from Swapnroop Drugs & Pharmaceuticals (Aurangabad, India) (Figure 1). Lecithin and sodium taurocholate were purchased from Alfa Aesar (Haverhill, MA, USA). FaSSIF-V2 medium was prepared according to Denninger et al. [30], in henceforth will be referred to as FaSSIF.

### 2.2. Supersaturation Assay

A supersaturation assay was conducted to assess the potential of several precipitation inhibitors (PVA, HPMCAS, copovidone, HPC-SSL, Eudragit L100-55, Kolliphor P188 and Kolliphor P407) to maintain the supersaturation of CXB. The experiments were performed using the MiniDissolution apparatus [31]. All experiments were conducted at 37 °C for 3 h at a paddle speed of 75 rpm. The dissolution medium consisted of 20 mL either plain PBS at pH 6.8 or PBS buffer containing dissolved precipitation inhibitor (0.18%). The supersaturation was induced by addition of 100 µL CXB stock solution in DMSO (40 mg/mL) to the dissolution medium, which equals a concentration of 200 µg/mL. The concentrations were determined online using an 8453 UV/VIS spectrophotometer (Agilent, Waldbronn, Germany) including correction for scattering.

### 2.3. Dynamic Light Scattering (DLS)

The evolution of the particle size of the precipitates from the supersaturation assay was monitored using a SZ-100 Nanoparticle Analyzer (Horiba, Kyoto, Japan) at a fixed angle of 90° and 37 °C. Samples were withdrawn from the supersaturation assay after 0, 30, 60, 120 and 180 min. The determined sizes are given as z-average value.

### 2.4. Hot-Melt Extrusion

The preparation of the ASDs was carried out using a 12 mm co-rotating twin screw extruder ZE 12 (Three-Tec GmbH, Seon, Switzerland) with a functional length of 25:1 L/D and five heating zones, equipped with a 2 mm die. The ASDs were prepared with a drug load of 10% and 20%, respectively. Additionally, a ternary ASD was prepared (7.2% CXB, 28.5% PVA, 64.3% HPMCAS). The composition was selected based on monophasic dissolution tests (see Section 3.5) The selected temperature for the preparation of the HPMCAS-based ASD was 140 °C and 190 °C for the PVA-based and ternary ASDs (except the initial heating zone, which was set to 100 °C in both cases). The feed-rate and screw speed was kept constant at 2 g/min and 100 rpm, respectively. The resulting extrudate strands were ground using a 400 MM mixer mill (Retsch, Haan Germany) and subsequently passed through a 355 µm mesh to remove large particles. 

### 2.5. Differential Scanning Calorimetry (DSC)

DSC measurements were conducted using a DSC 2 instrument (Mettler, Gießen, Germany equipped with nitrogen cooling system. Approximately 10 mg were accurately weighed into an aluminum pan with a pierced lid. The glass transition temperatures of the ASDs were determined using a temperature-modulated program (TOPEM-mode) with an underlying heating rate of 2 K/min with a nitrogen-purge of 30 mL/min.

### 2.6. X-Ray Powder Diffraction (XRPD)

XRPD studies were performed in reflection mode on an X’Pert MRD Pro (PANalytical, Almelo, The Netherlands). Nickel filtered CuKα1 radiation was generated at 45 kV and 40 mA and the scans were performed from 4 ° to 45 ° 2θ with a step size of 0.017 ° 2θ.

### 2.7. Fourier-Transform Infrared Spectroscopy (FT-IR)

Solid-state molecular interactions were studied using an Alpha FT-IR spectrometer (Bruker, Billerica, MA, USA) equipped with an attenuated total reflection (ATR) accessory. The spectral range was 400–4000 cm^−1^ and 24 scans were recorded each sample. All raw materials, physical mixtures and ASDs were measured.

### 2.8. Degree of Swelling and Erosion of the ASDs

The degree of swelling and the degree of erosion was determined for the pure polymers and for the ASDs. Therefore, the unmilled extrusion strands were manually cut into cylindrical shapes of approx. 30 mg to obtain macroscopic and weighable pieces. The cylindrical samples were placed in PBS buffer with pH of 6.8 and weighed after 3 h exposure to the buffer. The degree of swelling was determined according to Equation (1):

(1) $$Swelling\;\left(\%\right)=\frac{W_{s}-W_{i}}{W_{i}}\times 100$$

where *W_s_* is the weight of the swollen sample and *W_i_* is the weight of the initial sample. To determine the percentage of erosion the swollen sample was dried to constant mass. The calculation was performed using Equation (2):

(2) $$Erosion\;\left(\%\right)=\frac{W_{i}-W_{d}}{W_{i}}\times 100$$

where *W_d_* is the weight of the dried sample and *W_i_* is the weight of the initial sample.

### 2.9. Monophasic Non-Sink Dissolution

Non-sink dissolution experiments were conducted using the MiniDissolution apparatus with 20 mL medium at 37 °C and a paddle speed of 75 rpm for 3 h in total. Initially, the PVA-CXB and the HPMCAS-CXB ASDs (10% and 20% drug-load) in PBS at pH 6.8 or FaSSIF (adjusted to pH 6.8) were tested. The PVA ASDs were also tested in presence of predissolved HPMCAS in various concentrations or in combination with HPMCAS powder. The CXB concentration was determined online using an Agilent 8453 UV/VIS spectrophotometer. As there was an overlapping of the CXB and FaSSIF absorbance spectrum at the absorbance maximum of CXB (250 nm), the first derivative of the absorbance spectrum was used at 270 nm for quantification of CXB. Please see Supplementary Data Figure S1.

### 2.10. Biphasic Dissolution

Biphasic dissolution was carried out using the BiPha+ apparatus, which was previously developed in our group [30]. Briefly, 50 mL of aqueous phase (FaSSIF adjusted to pH 6.8) was placed in the dissolution vessel at a temperature of 37 °C. Subsequently, the formulation to be tested was added to the aqueous phase and after 30 min 1-decanol was overlaid onto the aqueous layer. The overall duration was 270 min and the concentration was recorded in the water phase and in the organic phase using an Agilent 8453 UV/VIS spectrophotometer.

## 3. Results

### 3.1. Supersaturation Assay

**Figure 2:**
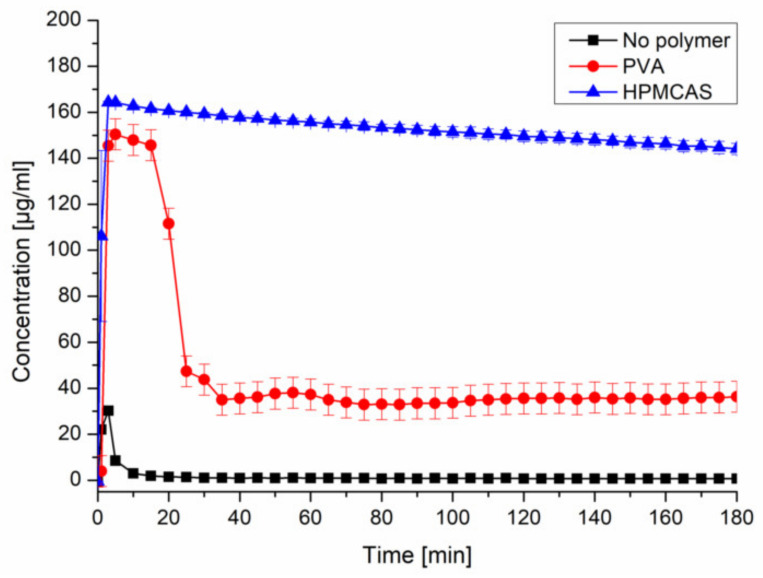
Supersaturation assay of CXB in PBS at pH 6.8 and in presence of predissolved PVA or HPMCAS (0.18%).

Upon inducing supersaturation of CXB without presence of a polymer, an immediate and complete precipitation could be observed. The presence of predissolved HPMCAS (0.18%) provided a pronounced and stable supersaturation (~150 µg/mL). The presence of predissolved PVA (0.18%) led to a comparable supersaturation within the first 20 min, followed by a severe drop to ~35 µg/mL (Figure 2). The selected concentration of CXB and polymer would equal a formulation with 10% drug load. The other tested precipitation inhibitors can be found in the Supplementary Data (Figure S2). 

### 3.2. Evolution of the Particle Size upon Supersaturation

**Figure 3:**
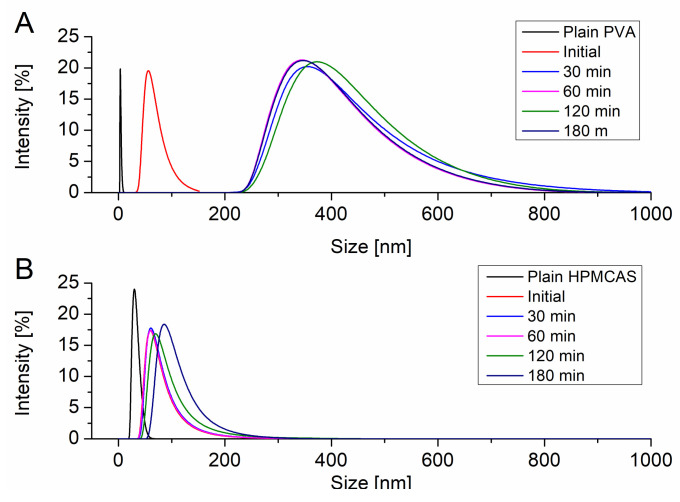
Evolution of the particle size upon supersaturation in presence of PVA (**A**) and HPMCAS (**B**).

The particle size of the resulting precipitates upon induced supersaturation was measured using DLS. All particle size distributions were monomodal. Figure 3A shows the evolution of the particle size of the precipitate in presence of dissolved PVA. PVA itself exhibited a hydrodynamic radius of 10 nm. Immediately after induction of the supersaturation in presence of PVA particles with a size of 68.1 nm could be detected. After 30 min the precipitate evolved to a larger size of 382.5 nm. In the further course of the supersaturation assay the size of the precipitate did not change and stayed in range of approx. 400 nm. HPMCAS itself showed a hydrodynamic radius of 30.5 nm. Upon supersaturation induction colloids of 69.9 nm were observed. These colloids remained in size dimensions of 100 nm without significant growth throughout the supersaturation assay (Figure 3B). 

### 3.3. Solid-State of the ASDs

**Figure 4:**
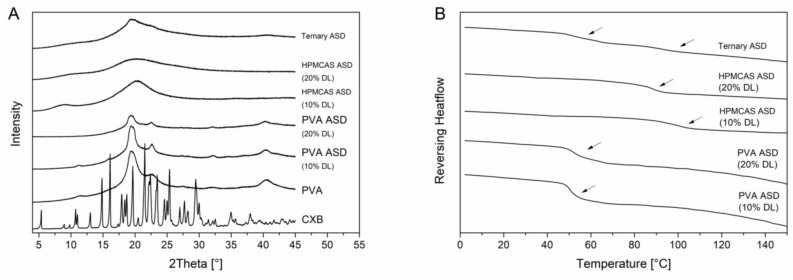
Solid state of the ASDs. X-ray powder diffraction (XRPD) diffractograms (**A**). Differential scanning calorimetry (DSC) thermograms (**B**), glass transition temperatures are indicated by the arrows.

PVA is a semi-crystalline polymer and it exhibited a glass transition temperature at 65.1 °C and a melting point at 170 °C. The semi-crystalline nature was also visible in the XRPD diffractogram (Figure 4B) since in addition to the amorphous halo there were also reflection peaks visible at 19.5° 2θ, 22.6° 2θ and 40.5° 2θ. The diffractograms of the PVA ASDs exhibited reflection peaks originated by the semi-crystalline structure of the PVA. However, there were no reflection peaks visible which could be related to CXB reflection peaks which indicated the CXB was transformed to the amorphous state (Figure 4A). The DSC measurements of the PVA ASDs (10% and 20% drug load) showed single glass transition temperatures of 51.1 ± 0.2 °C and 53.7 ± 0.3 °C, respectively. (Figure 4B). The HPMCAS ASDs (10% and 20% drug load) showed a completely amorphous halo and single glass transition of 96.7 ± 0.5°C and 87.8 ± 0.5 °C (Figure 4). Within the ternary ASD, CXB was also present in its amorphous form and two distinct glass transition temperatures were present at 55.0 ± 0.5 °C and 92.5 ± 0.4 °C, respectively. This observation showed that HPMCAS and PVA were not miscible and that CXB distributed within both polymers, since both glass transition temperatures related to the ones from the pure polymers shifted towards lower temperatures. 

**Figure 5:**
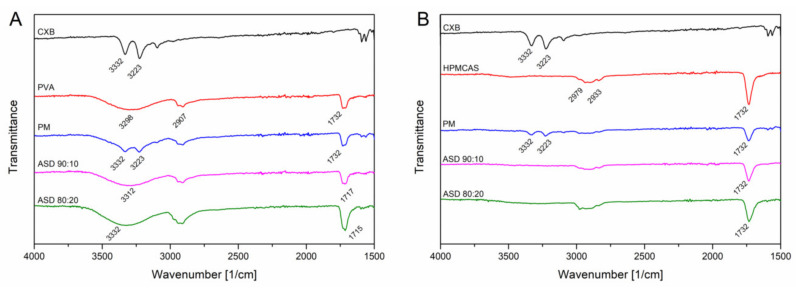
Fourier-transformed infrared spectra of CXB-PVA formulations (**A**) and CXB-HPMCAS formulations (**B**).

The FT-IR measurements for CXB showed NH stretching vibrations at 3332 cm^1^ and 3223 cm^−1^, an asymmetric and symmetric S=O stretching at 1346 cm^−1^ and 1167 cm^−1^, respectively and a CF stretching at 1230 cms^−1^ (Figure 5). For the PVA polymer a broad OH stretching at 3298 cm^−1^, CH stretching at 2907 cm^−1^ and a C=O stretching at 1732 cm^−1^ (related to the unhydrozyled PVAc) could be recorded. HPMCAS showed a OCH_3_ vibration at 2979 cm^−1^ and 2933 cm^−1^ and at 1732 cm^−1^ a C=O stretching vibration. For the PVA ASDs (10% and 20% drug load), the C=O peak shifted to 1717 cm^−1^ and 1715 cm^−1^, respectively. The broad OH band shifted to 3312 cm^−1^ and 3332 cm^−1^, respectively. The NH peak of CXB completely disappeared for the PVA ASDs. These observations clearly indicate the formation of hydrogen bonds between PVA and CXB. The C=O of the HPMCAS based ASDs did not shift compared to the neat HPMCAS spectrum, indicating that the C=O group was not involved into hydrogen bonds with CXB. However, the NH vibration peak from CXB completely vanished for the HPMCAS ASDs, which indicated presence of molecular interactions between CXB and HPMCAS. For the ternary ASD, a vanishing of the NH vibration peak occurred, too. In the C=O region a double peak could be observed, which was related to the respective C=O peaks from the pure polymers. Due to that double peak no precise assertions can be made with regard to peak shifting. The spectrum of the ternary ASD in a more narrow range can be found in Supplementary Data (Figure S3) together with the other ASDs. 

**Figure 6:**
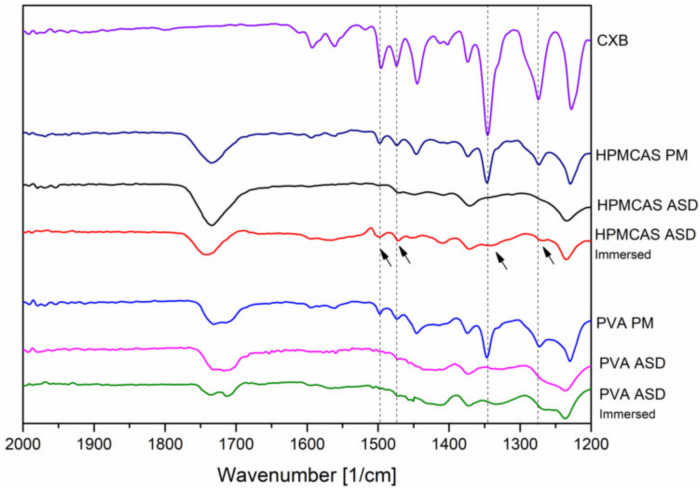
Fourier-transformed infrared spectra of the 20% drug load ASD after preparation and after immersion with buffer.

In addition, FT-IR spectra of the 20% drug load ASDs were recorded after immersion with buffer solution (for 15 min) and subsequent drying to investigate the phase behavior and interactions upon contact with buffer medium. After exposure of buffer medium, for the HPMCAS ASD some specific peaks from CXB appeared again, indicated by the arrows in Figure 6. 

Among these reappeared peaks was the stretching vibration of the hydrogen acceptor S=O group. In contrast, no change in the FT-IR spectrum of the PVA ASD could be observed upon immersion with buffer. 

### 3.4. Degree of Swelling and Erosion

The degree of swelling and erosion of the placebo extrudates and the ASDs was assessed to investigate the influence of the presence of CXB within the ASD formulations on the dissolution behavior. As shown in Table 1, for the pure polymers PVA and HPMCAS negative values for the degree of swelling were obtained indicating that dissolution was predominant over swelling, which was also reflected in a high degree of “erosion” (54.4% and 49.1%). Contrarily, the CXB-containing PVA ASDs exhibited a degree of swelling of 65.8% and 88.0% for the drug loads of 10% and 20%, respectively. The degree of erosion was also dependent on the drug load, whereby an increased drug load led to a decreased degree of erosion. For the HPMCAS ASDs a similar trend could be observed, but the extent of swelling was much more pronounced (170.8% and 175.4%), while the extent of erosion was markedly lower as compared to the PVA ASDs. For the ternary ASD the extent of swelling was 224.8% and the degree of erosion was at 6.7%. 

### 3.5. Monophasic Non-Sink Dissolution

In order to evaluate the performance from the monophasic dissolution experiment, the AUC of the supersaturation assay in presence of predissolved HPMCAS was calculated (27380 µg*min*mL^−1^) and taken as reference (i.e., maximum achievable performance). Upon dissolution of an ASD formulation, 100% of this AUC could only be achieved for an infinitely fast dissolution rate and absence of any precipitation and is therefore practically impossible to achieve. However, the more an AUC approaches this maximum value the better the performance of an ASD could be rated. In the following the ratio of a respective AUC to the maximum achievable AUC will be referred to as relative AUC (Table 2). 

#### 3.5.1. PVA ASDs in Presence of Predissolved HPMCAS

**Figure 7:**
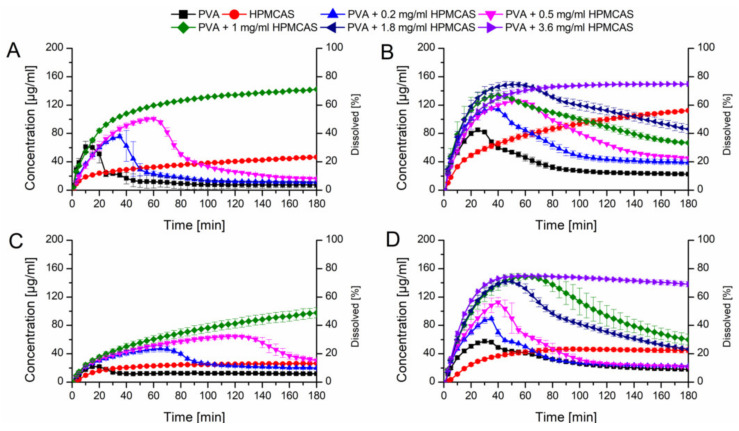
Monophasic non-sink dissolution studies of plain ASDs and of PVA ASDs in presence of predissolved HPMCAS at a drug load of 10% in PBS at pH 6.8 (**A**), in FaSSIF at pH 6.8 (**B**), at a drug load of 20% in PBS at pH 6.8 (**C**) and in FaSSIF at pH 6.8 (**D**).

Initially, the HPMCAS and PVA ASDs at a drug load of 10% without additives were tested in PBS buffer at pH 6.8 (Figure 7A). The HPMCAS ASD showed a slow dissolution rate ending up at 40 µg/mL and the relative AUC was 23.1%. The PVA ASD showed a fast dissolution rate reaching a concentration of 60 µg/mL in 10 min, which was followed by a precipitation over time, resulting in a relative AUC of 9.9%. 

Owing to the fact, that HPMCAS showed a high potential to stabilize supersaturated CXB, it was decided to test the dissolution performance of the fast dissolving PVA-CXB ASD in presence of various HPMCAS concentrations. In presence of 0.2 mg/mL predissolved HPMCAS, the dissolution rate of the PVA ASD (10% drug load) was comparable to the performance without presence HPMCAS, but the induction of precipitation was delayed. Increasing concentrations of HPMCAS involved an enhanced dissolution and delayed precipitation (Figure 7A). The best performance for the 10% drug load PVA ASD was achieved in presence of 1 mg/mL predissolved HPMCAS as no precipitation occurred at all resulting in a relative AUC of 77.6% (Table 2). 

Subsequently, dissolution experiments for the ASDs at a drug load of 10% were also carried out in FaSSIF dissolution medium, to generate more biorelevant circumstances and to study the influence of the dissolution medium on the required HPMCAS concentration for optimal dissolution performances. The dissolution performance of the HPMCAS and PVA ASDs was improved and entailed relative AUCs of 54.7% and 25.1%, respectively (Figure 7B). For the PVA formulation a more pronounced initial dissolution and also a more pronounced parachute effect was observed in presence of FaSSIF. The parachute effect for the PVA ASD could be further enhanced by the presence of predissolved HPMCAS. The relative AUC increased with increasing HPMCAS concentration. Interestingly, at a concentration of 1 mg/mL there was still a decrease in concentration over time i.e., precipitation visible, despite the fact that 1 mg/mL predissolved HPMCAS was sufficient to avoid precipitation in PBS. Therefore, the HPMCAS concentration was further increased until no concentration drop occurred, which was the case for a HPMCAS concentration of 3.6 mg/mL. The dissolution upon that conditions resulted in a relative AUC of 87.7% (Table 2).

These experiments were also conducted for ASDs containing 20% drug load. Due to the increased drug load, the dissolution performance was inferior compared to the 10% drug loaded ASDs (Figure 7C). The PVA and HPMCAS ASDs exhibited relative AUCs of 8.5% and 14.7%, respectively in plain buffer. Again, the dissolution of the PVA ASD could be enhanced in presence of HPMCAS, which was concentration dependent. At 1 mg/mL HPMCAS concentration no precipitation could be observed. However, the relative AUC of the 20% drug load ASD was lower than the one with 10% drug load (44.6% vs. 77.6%). Using FaSSIF as dissolution medium resulted in increased relative AUCs of the HPMCAS and PVA ASDs (26.0% and 20.4%), respectively compared to the experiments conducted in plain buffer as already observed for the ASDs containing 10% CXB (Figure 7D). Just as previously observed, the dissolution performance of the PVA ASD could be enhanced by the presence of predissolved HPMCAS. The concentration effects were comparable with the data obtained for the 10% drug load ASD, whereby the relative AUC increased with increased HPMCAS concentration with a maximum relative AUC of 88.8% at 3.6 mg/mL.

#### 3.5.2. PVA ASDs with Physically Added HPMCAS Powder and Ternary ASD

**Figure 8:**
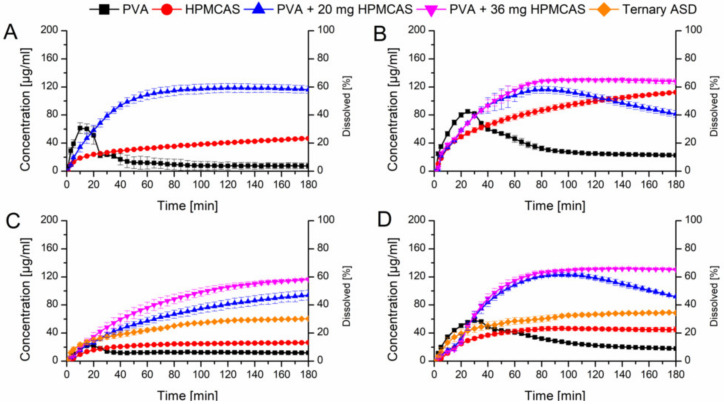
Monophasic non-sink dissolution studies of plain ASDs and of PVA ASDs with admixed HPMCAS powder at a drug load of 10% in PBS at pH 6.8 (**A**), in FaSSIF at pH 6.8 (**B**), at a drug load of 20%, including ternary ASD in PBS at pH 6.8 (**C**) and in FaSSIF at pH 6.8 (**D**).

In a next step it was verified if the beneficial effect of the predissolved HPMCAS on the dissolution of the PVA ASDs would also appear if HPMCAS was added as powder. Two levels (20 mg or 36 mg) of HPMCAS were physically added to the PVA ASDs which equals a concentration of 1.0 mg/mL and 1.8 mg/mL HPMCAS, respectively derived from the previous experiments. Figure 8A shows the comparison of the HPMCAS and PVA ASDs at a drug load of 10% in plain buffer and the PVA ASD with 20 mg HPMCAS powder. The addition of 20 mg HPMCAS powder had a significant impact on the dissolution profile of the PVA-CXB ASD. The maximum concentration (120 µg/mL) was increased 15-fold compared to the plain PVA ASD and the relative AUC was 6.7-fold increased (66.5% vs. 9.9%). As previous, these experiments were repeated with FaSSIF as medium (Figure 8B). The addition of 20 mg HPMCAS showed a very similar initial dissolution profile as the one in plain buffer, but after 80 min there was an onset of precipitation observable. Therefore, the HPMCAS amount was increased to 36 mg equivalent to 1.8 mg/mL. At this level no precipitation occurred and a relative AUC of 70.2% was achieved.

Adding 20 mg HPMCAS powder to the PVA ASD with 20% drug load in buffer medium resulted in very comparable results to the experiment with predissolved HPMCAS (42.3% vs. 44.6%) (Figure 8C). Increasing the amount to 36 mg HPMCAS even enhanced the relative AUC to 54.6%, in both cases precipitation was absent. Again, when FaSSIF was used as dissolution medium at a level of 20 mg HPMCAS addition to the PVA ASD at 20% drug load, precipitation occurred after 100 min. With addition of 36 mg HPMCAS powder no precipitation occurred and a relative AUC of 69.6% could be obtained (Figure 8D). 

Based on the optimized amount of HPMCAS, also a ternary mixture was melt extruded (the composition was equivalent to the 20% PVA ASD with 36 mg HPMCAS powder). The relative AUC from the dissolution experiments in buffer and FaSSIF (31.4% and 48.4%) were higher as compared to the plain binary ASDs, but distinctly lower compared to the optimized PVA ASD with admixed HPMCAS powder.

### 3.6. Biphasic Dissolution

**Figure 9:**
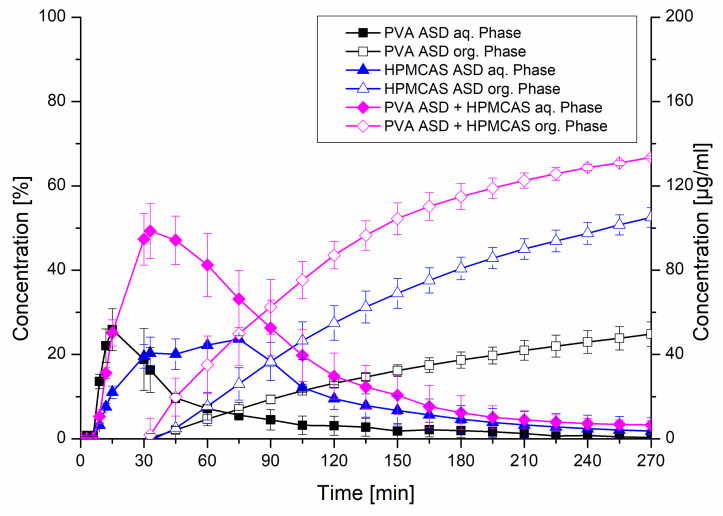
Biphasic dissolution studies of plain ASDs and the PVA ASD with admixed HPMCAS powder at a drug load of 20%. The aqueous medium consisted of FaSSIF adjusted to pH 6.8 and the organic phase was 1-decanol.

The formulations exhibiting 20% drug load including the HPMCAS ASD, the PVA ASD and the PVA ASD with HPMCAS powder (equivalent to a HPMCAS concentration of 1.8 mg/mL) were further investigated in the biphasic dissolution test using FaSSIF as biorelevant medium. In the aqueous phase, the PVA ASD showed a similar initial dissolution behavior compared to the monophasic test. The HPMCAS ASD showed a more moderate dissolution, which reached a plateau after approx. 40 min, which was in accordance to the monophasic dissolution. The most pronounced dissolution was observed for the PVA ASD with HPMCAS powder, which reached a concentration of 50 µg/mL after 30 min, comparable to the monophasic test. Subsequently, a drop in concentration in the aqueous phase occurred, which was related to the addition of the organic layer after 30 min, because the dissolved CXB started partitioning into the organic layer. The partitioning rates of the three tested formulations into the organic phase were in accordance to the rank order of the dissolution in the aqueous phase. The combination of the PVA ASD with the HPMCAS powder resulted in a partitioned amount of 66.8%, whereas final partitioned amount of CXB from the PVA ASD was only 24.8%. Consequently, the addition of the HPMCAS powder to the PVA ASD led to a 2.7-fold increased partitioned amount. The biphasic dissolution of the HPMCAS ASD resulted in a partitioned amount of 52.5% (Figure 9).

## 4. Discussion

The supersaturation assay revealed a good precipitation inhibitory behavior of HPMCAS for CXB. As revealed by DLS measurements, nanoscale particles or colloids in a size range of 100 nm were present upon induction of the supersaturation. HPMCAS functioned as effective growth inhibitor as the particle size did not grow over time, while a high level of supersaturation could be maintained. Ueda et al. showed that the presence of those nanoscale colloids could indicate the formation of liquid-liquid phase separation [32]. This phenomenon describes a phase transition of a supersaturated solution into a drug-rich and solvent-rich phase [33]. The occurrence of nanoscale colloids or liquid-liquid phase separation can act as a reservoir for the supersaturation level as a result of rapid re-dissolution kinetics and it is also related to an enhanced absorption [33,34,35]. Consequently, the occurrence of that phenomenon can be considered as beneficial for an ASD upon dissolution. For HPMCAS, mainly hydrophobic interactions were discussed as predominant interaction mechanism for its precipitation inhibitory potential [36,37]. Ilevbare et al. showed a correlation between hydrophobicity of a polymer and its potential to inhibit nucleation and crystal growth of a supersaturated drug [7,8]. The solubility parameter was taken as indicator for the degree of hydrophobicity and it was hypothesized that it would be favorable if the polymer and the drug have similar solubility parameters. The fact that the solubility parameters of CXB and HPMCAS (23.4 MPa^1/2^ and 22.4 MPa^1/2^) are very close to each other confirms this hypothesis, which is also in line with the data in our study. 

In contrast, PVA was only able to maintain the highly supersaturated state for 20 min. The DLS measurements revealed that the nanocolloids increased in size over time from below 100 nm to approx. 400 nm. Hence, it can be concluded that PVA was a less effective stabilizer for the size of the nanocolloids, which in turn reduces re-dissolution of those. Further, contemplating the solubility parameter of PVA (32.4 MPa^1/2^), it suggests a very pronounced hydrophilicity rather than hydrophobicity. According to the study of Ilevbare et al. this high discrepancy between the solubility parameter of CXB and PVA (i.e., high difference in hydrophobicity) is considered unconducive when it comes to inhibition of nucleation and crystal growth [8]. It has been discussed that polymers exhibiting a high hydrophilicity tend to preferably interact with water, which is also highly hydrophilic rather than with the hydrophobic drug substance [38]. Chen et al. emphasized that interactions between a highly hydrophilic polymer with a hydrophobic drug are “water-vulnerable”, which means that the interactions between the polymer and the drug become weaker over time, while the interactions between the polymer and water become stronger [16]. This fact is perfectly in line with our data from the supersaturation assay, where the supersaturation in presence of PVA dramatically crashed after a certain amount of time. 

Despite the high potential of HPMCAS to stabilize supersaturated CXB, the ASD formulations containing HPMCAS did not achieve as high concentration levels upon dissolution. While pure HPMCAS solely tended to dissolve instead of swell, the situation profoundly changed for the ASDs containing CXB, which exhibited a high degree of swelling and a low degree of erosion. As reported by Han et al. and Saboo et al. this could be a result of incongruent release of polymer and drug from the ASD caused by water-induced phase separation [39,40]. The FT-IR spectra after buffer treatment indicated an AAPS of CXB and HPMCAS or at least revealed a weakening of the interactions between drug and polymer. As a consequence of the weakened interactions and the high degree of swelling instead of dissolution, only low levels of supersaturation were achieved for the HPMCAS ASDs. On the contrary, PVA ASDs showed a lower degree of swelling and a higher degree of erosion. Also, there was no phase separation or weakening of the interactions between CXB and PVA observable upon immersion with buffer medium. Consequently, the dissolution rate was markedly higher compared to the HPMCAS ASDs. It has been shown that ASD formulations using PVA as carrier exhibited relatively higher dissolution rates compared to other common carriers [41,42]. In order to benefit from both, a rapid dissolution and a sufficiently stabilized supersaturation, it was decided to combine the PVA ASD with predissolved or physically added HPMCAS and additionally, a ternary ASD was formulated. The monophasic dissolution experiments of the PVA ASDs in PBS showed that an increase of the predissolved HPMCAS concentration led to an increased dissolution and supersaturation performance, whereby 1 mg/mL HPMCAS provided the best performance. When using FaSSIF as dissolution medium, higher HPMCAS concentrations were required to obtain optimal dissolution and supersaturation performance. This fact is likely attributed to competitive interactions between HPMCAS, the surfactants from the FaSSIF medium and CXB. Pinto et al. (2019) emphasized the relevance of biorelevant media, as they revealed specific interactions between polymer and surfactants, which led to a decreased supersaturation performance in FaSSIF compared to plain buffer [43]. In line with this, Pui et al. also found a decreased supersaturation ability of a polymer due to competitive interactions with surfactants [44]. Consequently, the HPMCAS concentration needed to be increased to 3.6 mg/mL in presence of FaSSIF. 

In order to estimate if this synergism of PVA and HPMCAS is conveyable to a solid formulation approach, HPMCAS powder was admixed with the PVA ASD. The monophasic dissolution experiments showed very similar dissolution profiles and the rel. AUCs were in the same range. Interestingly, when FaSSIF medium was used, only an amount of HPMCAS equivalent to 1.8 mg/mL was necessary to avoid precipitation, which is 2-fold less compared to the experiment with predissolved HPMCAS. Since the dissolution in presence of predissolved HPMCAS was more rapid, the maximum level of supersaturation was reached faster. In this respect, it was shown that the supersaturation generation rate remarkably influences the precipitation kinetics [45]. 

The optimized composition was also formulated as ternary ASD. The monophasic dissolution performance was slightly better compared to the binary ASDs but could not reach as high concentrations as the PVA ASDs with physically added HPMCAS powder. For the ternary ASD, the DSC measurement revealed two distinct glass transition temperatures, which indicated an immiscibility of HPMCAS and PVA. This fact might have contributed to the increased degree of swelling of the ternary system. Consequently, the pronounced degree of swelling and limited degree of erosion led to an incomplete dissolution. 

Finally, the optimized formulation composition for the 20% drug load PVA ASD with admixed HPMCAS powder and the binary ASDs (20% drug load) were also assessed in a biphasic dissolution test. It has been shown that drug-polymer interactions can alter the absorption rate, therefore monophasic dissolution tests are not always sufficiently predictive for absorption [46]. It has been revealed that the thermodynamic activity is more important for the flux or the absorption than the apparent concentration [47]. Therefore, it is desirable to introduce a dissolution model with an absorptive compartment. Biphasic dissolution models showed excellent in-vivo predictability for poorly soluble compounds and formulations thereof [48,49,50]. Shi et al. showed a linear relationship between the end-concentration in the organic phase and the relative bioavailability of different formulations for a biphasic dissolution model [51]. Therefore, the results from the biphasic dissolution experiments suggest a significantly higher absorption rate and therefore ultimately an increased bioavailability of the PVA ASD combined with HPMCAS powder compared to the plain ASDs. When it comes to a final formulation of a dosage form, it should be considered that HPMCAS exhibits a pH-dependent solubility, hence it is insoluble in acidic pH which is present in the gastric stage. Consequently, in order to ensure simultaneous dissolution of HPMCAS and the PVA ASD, it is suggested to generate an enteric final dosage form like enteric capsules or tablets with an enteric coating. 

## 5. Conclusions

In this study it was shown that HPMCAS exhibits a higher potential to stabilize supersaturated CXB solutions compared to PVA as a consequence of their similar hydrophobicity. The subsequently prepared binary CXB ASDs with either HPMCAS or PVA as matrix polymer were of amorphous nature. When formulated as ASD, the HPMCAS systems showed a suppressed dissolution behavior as a result of a high degree of swelling and weakened interactions between CXB and HPMCAS, which led to a high unexploited potential with regard to the degree of supersaturation. On the contrary, the PVA ASDs exhibited a high dissolution rate facilitated by the hydrophilic nature of the polymer and stronger hydrogen bonds with CXB within the solid phase, but with a subsequent precipitation over time. With HPMCAS being predissolved in the dissolution medium, the dissolution performance of the PVA ASDs could be markedly improved as a function of HPMCAS concentration. Derived from the results with predissolved HPMCAS, a solid formulation could be generated which showed similarly good dissolution performances with a stable and high level of supersaturation. The PVA ASD with physically admixed HPMCAS powder was also superior in a biorelevant biphasic dissolution model compared to the binary ASDs. Conclusively, CXB was successfully formulated as supersaturable formulation by generating an ASD with a high dissolution rate combined with a suitable precipitation inhibitor. 

## Figures and Tables

**Table 1:** Degree of swelling and erosion of placebo extrudates and ASDs (*n* = 3).

Extrudate	Swelling (%)	Erosion (%)
PVA	–41.1 ± 0.8	54.4 ± 1.2
PVA ASD (10%)	65.8 ± 4.7	18.9 ± 0.9
PVA ASD (20%)	88.0 ± 4.6	2.8 ± 0.1
HPMCAS	–41.6 ± 3.2	49.1 ± 3.3
HPMCAS ASD (10%)	170.8 ± 3.0	7.3 ± 1.8
HPMCAS ASD (20%)	175.4 ± 3.5	1.1 ± 1.6
Ternary ASD	224.8 ± 7.4	6.7 ± 1.1

**Table 2:** Relative area under the dissolution curve.

Formulation/Composition	10% DL		20% DL	
	Buffer	FaSSIF	Buffer	FaSSIF
HPMCAS ASD	23.1 ± 0.6%	54.7 ± 2.2%	14.7 ± 0.4%	26.0 ± 1.7%
PVA ASD	9.9 ± 2.3%	25.1 ± 1.0%	8.5 ± 0.6%	20.4 ± 0.3%
PVA ASD + 0.2 mg/mL HPMCAS	16.3 ± 1.8%	41.4 ± 2.2%	19.1 ± 1.0%	25.2 ± 0.4%
PVA ASD + 0.5 mg/mL HPMCAS	30.5 ± 0.4%	52.3 ± 0.1%	31.1 ± 0.2%	31.3 ± 0.3%
PVA ASD + 1.0 mg/mL HPMCAS	77.6 ± 0.1%	63.3 ± 0.6%	44.6 ± 3.6%	67.0 ± 3.2%
PVA ASD + 1.8 mg/mL HPMCAS	n.d.	75.3 ± 3.6%	n.d.	56.2 ± 0.9%
PVA ASD + 3.6 mg/mL HPMCAS	n.d.	87.7 ± 2.2%	n.d.	88.8 ± 1.2%
PVA ASD + 20 mg HPMCAS powder (≙ 1.0 mg/mL)	66.5 ± 4.0%	58.5 ± 3.0%	42.3 ± 2.4%	62.1 ± 0.1%
PVA ASD + 36 mg HPMCAS powder (≙ 1.8 mg/mL)	n.d.	70.2 ± 2.0%	54.6 ± 3.3%	69.6 ± 1.5%
Ternary ASD	-	-	31.4 ± 0.8%	48.4 ± 2.0%

## Supplementary Materials

The following are available online at https://www.mdpi.com/1999-4923/12/6/541/s1, **Figure S1**: UV absorbance spectra of CXB (80 µg/mL in MeOH), PVA (1 mg/mL in PBS), HPMCAS (1 mg/mL in PBS) and FaSSIF (top graph) and the respective first derivative of the absorbance spectra (bottom graph); Figure S2: Supersaturation assay of CXB in PBS at pH 6.8 and in presence of various predissolved precipitation inhibitors (0.18%); Figure S3: FT-IR spectra of celecoxib, physical mixtures and amorphous solid dispersions including the ternary ASD.

## References

[1] Breitenbach J. (2002). Melt extrusion: From process to drug delivery technology. Eur. J. Pharm. Biopharm..

[2] Chiou W.L., Riegelman S. (1971). Pharmaceutical Applications of Solid Dispersion Systems. J. Pharm. Sci..

[3] Okonogi S., Oguchi T., Yonemochi E., Puttipipatkhachorn S., Yamamoto K. (1997). Improved dissolution of ofloxacin via solid dispersion. Int. J. Pharm..

[4] Van den Mooter G. (2012). The use of amorphous solid dispersions: A formulation strategy to overcome poor solubility and dissolution rate. Drug Discov. Today Technol..

[5] Lin X., Hu Y., Liu L., Su L., Li N., Yu J., Tang B., Yang Z. (2018). Physical Stability of Amorphous Solid Dispersions: A Physicochemical Perspective with Thermodynamic, Kinetic and Environmental Aspects. Pharm. Res..

[6] Chokshi R.J., Zia H., Sandhu H.K., Shah N.H., Malick W.A. (2007). Improving the Dissolution Rate of Poorly Water Soluble Drug by Solid Dispersion and Solid Solution—Pros and Cons. Drug Deliv..

[7] Ilevbare G.A., Liu H., Edgar K.J., Taylor L.S. (2012). Understanding Polymer Properties Important for Crystal Growth Inhibition—Impact of Chemically Diverse Polymers on Solution Crystal Growth of Ritonavir. Cryst. Growth Des..

[8] Ilevbare G.A., Liu H., Edgar K.J., Taylor L.S. (2013). Maintaining Supersaturation in Aqueous Drug Solutions: Impact of Different Polymers on Induction Times. Cryst. Growth Des..

[9] Chen Y., Wang S., Wang S., Liu C., Su C., Hageman M., Hussain M., Haskell R., Stefanski K., Qian F. (2016). Initial Drug Dissolution from Amorphous Solid Dispersions Controlled by Polymer Dissolution and Drug-Polymer Interaction. Pharm. Res..

[10] Sarode A.L., Sandhu H., Shah N., Malick W., Zia H. (2013). Hot melt extrusion (HME) for amorphous solid dispersions: Predictive tools for processing and impact of drug–polymer interactions on supersaturation. Eur. J. Pharm. Sci..

[11] Rumondor A.C.F., Wikström H., Van Eerdenbrugh B., Taylor L.S. (2011). Understanding the Tendency of Amorphous Solid Dispersions to Undergo Amorphous–Amorphous Phase Separation in the Presence of Absorbed Moisture. AAPS PharmSciTech.

[12] Purohit H.S., Taylor L.S. (2017). Phase Behavior of Ritonavir Amorphous Solid Dispersions during Hydration and Dissolution. Pharm. Res..

[13] Indulkar A.S., Lou X., Zhang G.G.Z., Taylor L.S. (2019). Insights into the Dissolution Mechanism of Ritonavir–Copovidone Amorphous Solid Dispersions: Importance of Congruent Release for Enhanced Performance. Mol. Pharm..

[14] Purohit H.S., Taylor L.S. (2015). Phase Separation Kinetics in Amorphous Solid Dispersions Upon Exposure to Water. Mol. Pharm..

[15] Vasanthavada M., Tong W.-Q.T., Joshi Y., Kislalioglu M.S. (2005). Phase Behavior of Amorphous Molecular Dispersions II: Role of Hydrogen Bonding in Solid Solubility and Phase Separation Kinetics. Pharm. Res..

[16] Chen Y., Pui Y., Chen H., Wang S., Serno P., Tonnis W., Chen L., Qian F. (2019). Polymer-Mediated Drug Supersaturation Controlled by Drug–Polymer Interactions Persisting in an Aqueous Environment. Mol. Pharm..

[17] Van Ngo H., Nguyen P.K., Van Vo T., Duan W., Tran V.-T., Tran P.H.-L., Tran T.T.-D. (2016). Hydrophilic-hydrophobic polymer blend for modulation of crystalline changes and molecular interactions in solid dispersion. Int. J. Pharm..

[18] Xie T., Taylor L.S. (2016). Dissolution Performance of High Drug Loading Celecoxib Amorphous Solid Dispersions Formulated with Polymer Combinations. Pharm. Res..

[19] Yazdanian M., Briggs K., Jankovsky C., Hawi A. (2004). The “High Solubility” Definition of the Current FDA Guidance on Biopharmaceutical Classification System May Be Too Strict for Acidic Drugs. Pharm. Res..

[20] Xie T., Taylor L.S. (2016). Improved Release of Celecoxib from High Drug Loading Amorphous Solid Dispersions Formulated with Polyacrylic Acid and Cellulose Derivatives. Mol. Pharm..

[21] Lainé A.-L., Price D., Davis J., Roberts D., Hudson R., Back K., Bungay P., Flanagan N. (2016). Enhanced oral delivery of celecoxib via the development of a supersaturable amorphous formulation utilising mesoporous silica and co-loaded HPMCAS. Int. J. Pharm..

[22] Chavan R.B., Rathi S., Jyothi V.G.S.S., Shastri N.R. (2019). Cellulose based polymers in development of amorphous solid dispersions. Asian J. Pharm. Sci..

[23] Monschke M., Wagner K.G. (2019). Amorphous solid dispersions of weak bases with pH-dependent soluble polymers to overcome limited bioavailability due to gastric pH variability—An in-vitro approach. Int. J. Pharm..

[24] Zecevic D.E., Meier R., Daniels R., Wagner K.-G. (2014). Site specific solubility improvement using solid dispersions of HPMC-AS/HPC SSL—Mixtures. Eur. J. Pharm. Biopharm..

[25] Monschke M., Kayser K., Wagner K.G. (2020). Processing of Polyvinyl Acetate Phthalate in Hot-Melt Extrusion—Preparation of Amorphous Solid Dispersions. Pharmaceutics.

[26] Knopp M.M., Nguyen J.H., Becker C., Francke N.M., Jørgensen E.B., Holm P., Holm R., Mu H., Rades T., Langguth P. (2016). Influence of polymer molecular weight on in vitro dissolution behavior and in vivo performance of celecoxib:PVP amorphous solid dispersions. Eur. J. Pharm. Biopharm..

[27] Meng F., Ferreira R., Zhang F. (2019). Effect of surfactant level on properties of celecoxib amorphous solid dispersions. J. Drug Deliv. Sci. Technol..

[28] Brough C., Miller D.A., Keen J.M., Kucera S.A., Lubda D., Williams R.O. (2016). Use of Polyvinyl Alcohol as a Solubility-Enhancing Polymer for Poorly Water Soluble Drug Delivery (Part 1). AAPS PharmSciTech.

[29] Wlodarski K., Zhang F., Liu T., Sawicki W., Kipping T. (2018). Synergistic Effect of Polyvinyl Alcohol and Copovidone in Itraconazole Amorphous Solid Dispersions. Pharm. Res..

[30] Denninger A., Westedt U., Rosenberg J., Wagner K.G. (2020). A Rational Design of a Biphasic DissolutionSetup—Modelling of Biorelevant Kinetics for a Ritonavir Hot-Melt Extruded Amorphous Solid Dispersion. Pharmaceutics.

[31] Zecevic D.E., Wagner K.G. (2013). Rational Development of Solid Dispersions via Hot-Melt Extrusion Using Screening, Material Characterization, and Numeric Simulation Tools. J. Pharm. Sci..

[32] Ueda K., Higashi K., Moribe K. (2019). Mechanistic elucidation of formation of drug-rich amorphous nanodroplets by dissolution of the solid dispersion formulation. Int. J. Pharm..

[33] Indulkar A.S., Gao Y., Raina S.A., Zhang G.G.Z., Taylor L.S. (2016). Exploiting the Phenomenon of Liquid–Liquid Phase Separation for Enhanced and Sustained Membrane Transport of a Poorly Water-Soluble Drug. Mol. Pharm..

[34] Hate S.S., Reutzel-Edens S.M., Taylor L.S. (2019). Insight into Amorphous Solid Dispersion Performance by Coupled Dissolution and Membrane Mass Transfer Measurements. Mol. Pharm..

[35] Ueda K., Higashi K., Yamamoto K., Moribe K. (2015). Equilibrium State at Supersaturated Drug Concentration Achieved by Hydroxypropyl Methylcellulose Acetate Succinate: Molecular Characterization Using ^1^H NMR Technique. Mol. Pharm..

[36] Chavan R.B., Thipparaboina R., Kumar D., Shastri N.R. (2016). Evaluation of the inhibitory potential of HPMC, PVP and HPC polymers on nucleation and crystal growth. RSC Adv..

[37] Curatolo W., Nightingale J.A., Herbig S.M. (2009). Utility of Hydroxypropylmethylcellulose Acetate Succinate (HPMCAS) for Initiation and Maintenance of Drug Supersaturation in the GI Milieu. Pharm. Res..

[38] Ueda K., Higashi K., Yamamoto K., Moribe K. (2014). The effect of HPMCAS functional groups on drug crystallization from the supersaturated state and dissolution improvement. Int. J. Pharm..

[39] Han Y.R., Ma Y., Lee P.I. (2019). Impact of phase separation morphology on release mechanism of amorphous solid dispersions. Eur. J. Pharm. Sci..

[40] Saboo S., Mugheirbi N.A., Zemlyanov D.Y., Kestur U.S., Taylor L.S. (2019). Congruent release of drug and polymer: A “sweet spot” in the dissolution of amorphous solid dispersions. J. Control. Release.

[41] De Jaeghere W., De Beer T., Van Bocxlaer J., Remon J.P., Vervaet C. (2015). Hot-melt extrusion of polyvinyl alcohol for oral immediate release applications. Int. J. Pharm..

[42] Umemoto Y., Uchida S., Yoshida T., Shimada K., Kojima H., Takagi A., Tanaka S., Kashiwagura Y., Namiki N. (2020). An effective polyvinyl alcohol for the solubilization of poorly water-soluble drugs in solid dispersion formulations. J Drug Deliv. Sci. Technol..

[43] Pinto J.M.O., Rengifo A.F.C., Mendes C., Leão A.F., Parize A.L., Stulzer H.K. (2019). Understanding the interaction between Soluplus^®^ and biorelevant media components. Colloids Surf. B Biointerfaces.

[44] Pui Y., Chen Y., Chen H., Wang S., Liu C., Tonnis W., Chen L., Serno P., Bracht S., Qian F. (2018). Maintaining Supersaturation of Nimodipine by PVP with or without the Presence of Sodium Lauryl Sulfate and Sodium Taurocholate. Mol. Pharm..

[45] Sun D.D., Lee P.I. (2013). Evolution of Supersaturation of Amorphous Pharmaceuticals: The Effect of Rate of Supersaturation Generation. Mol. Pharm..

[46] Raina S.A., Zhang G.G.Z., Alonzo D.E., Wu J., Zhu D., Catron N.D., Gao Y., Taylor L.S. (2015). Impact of Solubilizing Additives on Supersaturation and Membrane Transport of Drugs. Pharm. Res..

[47] Elkhabaz A., Moseson D.E., Brouwers J., Augustijns P., Taylor L.S. (2019). Interplay of Supersaturation and Solubilization: Lack of Correlation between Concentration-Based Supersaturation Measurements and Membrane Transport Rates in Simulated and Aspirated Human Fluids. Mol. Pharm..

[48] Frank K.J., Locher K., Zecevic D.E., Fleth J., Wagner K.G. (2014). In vivo predictive mini-scale dissolution for weak bases: Advantages of pH-shift in combination with an absorptive compartment. Eur. J. Pharm. Sci..

[49] Heigoldt U., Sommer F., Daniels R., Wagner K.-G. (2010). Predicting in vivo absorption behavior of oral modified release dosage forms containing pH-dependent poorly soluble drugs using a novel pH-adjusted biphasic in vitro dissolution test. Eur. J. Pharm. Biopharm..

[50] O’Dwyer P.J., Imanidis G., Box K.J., Reppas C. (2020). On the Usefulness of Two Small-Scale In Vitro Setups in the Evaluation of Luminal Precipitation of Lipophilic Weak Bases in Early Formulation Development. Pharmaceutics.

[51] Shi Y., Erickson B., Jayasankar A., Lu L., Marsh K., Menon R., Gao P. (2016). Assessing Supersaturation and Its Impact on In Vivo Bioavailability of a Low-Solubility Compound ABT-072 With a Dual pH, Two-Phase Dissolution Method. J. Pharm. Sci..

