# Supplementary material for: Impact of HPMCAS on the Dissolution Performance of Polyvinyl Alcohol Celecoxib Amorphous Solid Dispersions
Source: Pharmaceutics. 2020 Jun 11;12(6):541. doi: 10.3390/pharmaceutics12060541 (PMC7356348; doi:10.3390/pharmaceutics12060541)
Supplement: Supplementary file 1 [file pharmaceutics-12-00541-s001.pdf]

pharmaceutics

MDPI

Supplementary Materials:

Impact of HPMCAS on the Dissolution Performance of Polyvinyl Alcohol Celecoxib Amorphous Solid Dispersions

Marius Monschke and Karl G. Wagner *

Department of Pharmaceutical Technology and Biopharmaceutics, University of Bonn, 53121 Bonn, Germany; marius.monschke@uni-bonn.de

* Correspondence: karl.wagner@uni-bonn.de; Tel.: +49-228-73-5271

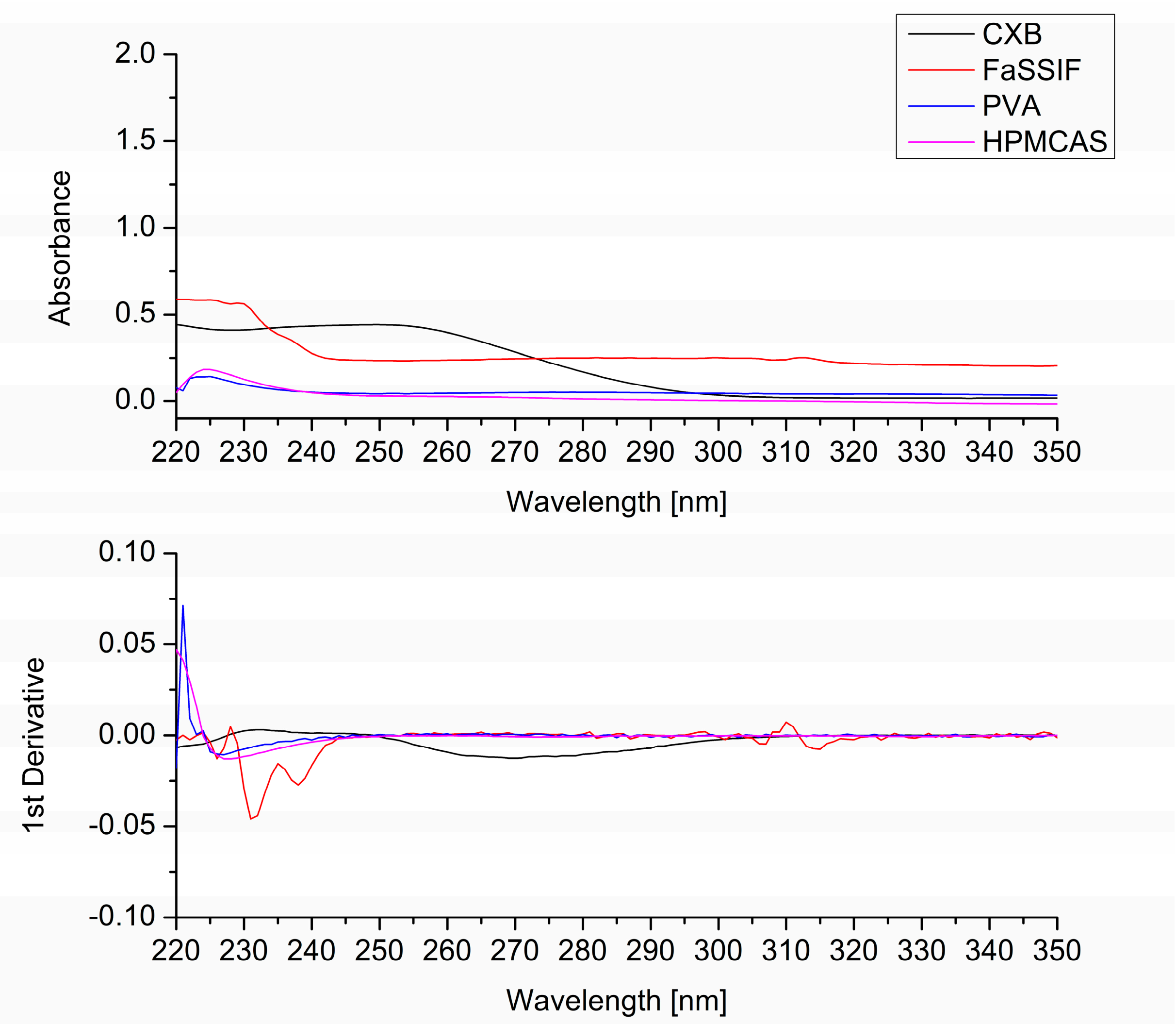

Figure S1. UV absorbance spectra of CXB (80 µg/mL in MeOH), PVA (1 mg/mL in PBS), HPMCAS (1 mg/mL in PBS) and FaSSIF (top graph) and the respective first derivative of the absorbance spectra (bottom graph).

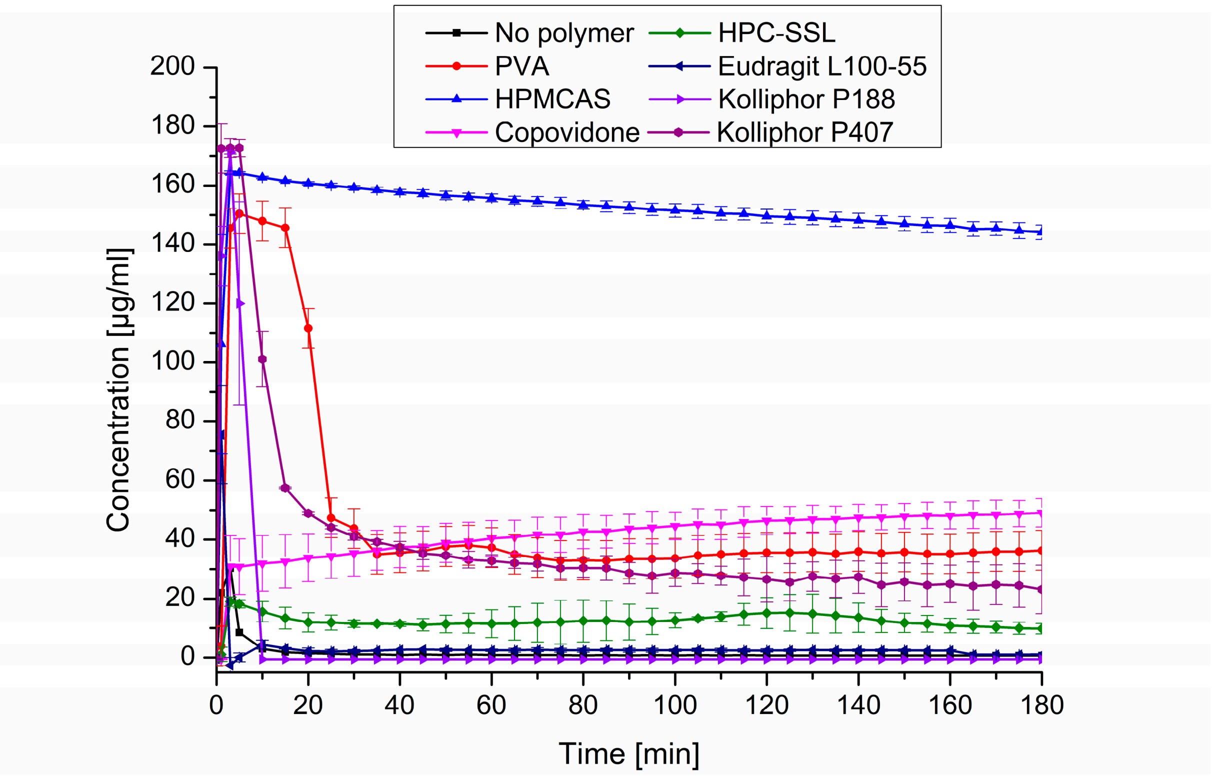

Figure S2. Supersaturation assay of CXB in PBS at pH 6.8 and in presence of various predissolved precipitation inhibitors (0.18%).

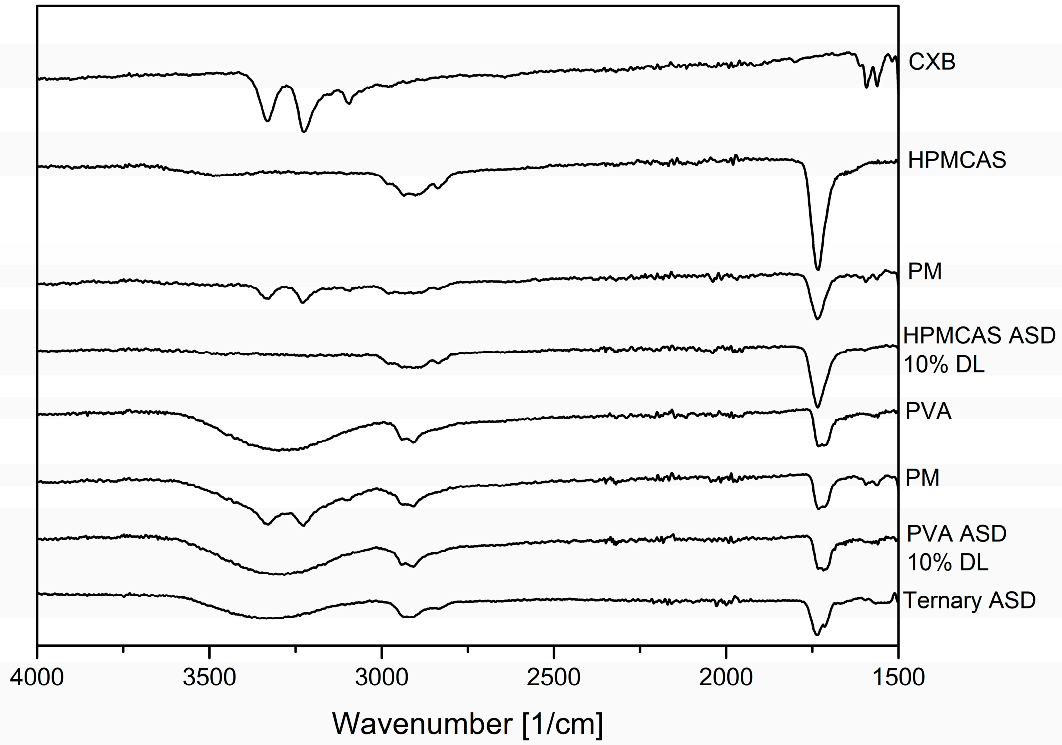

Figure S3. FT-IR spectra of celecoxib, physical mixtures and amorphous solid dispersions including the ternary ASD.
